# Husbandry factors are associated with skin, gastrointestinal and ophthalmic disorders in leopard geckos (*Eublepharis macularius*)

**DOI:** 10.1002/vetr.70971

**Published:** 2026-07-31

**Authors:** Joshua Henley, Tom Dutton, Joanna Hedley

**Affiliations:** ^1^ Taverham Veterinary Hospital Taverham UK; ^2^ The Avian Vet Gloucestershire UK; ^3^ Department of Clinical Science and Services, Beaumont Sainsbury Animal Hospital Royal Veterinary College London UK

## Abstract

**Background:**

Research into disorders in captive reptiles is notably sparse in the UK, despite the popularity of various species as pets. This study aimed to survey and investigate risk factors for disorders of leopard geckos in the UK.

**Methods:**

Medical records for leopard geckos presenting at three veterinary practices between 2019 and 2023 were reviewed retrospectively.

**Results:**

The medical records of 214 leopard geckos were included in the analyses. Non‐specific signs such as reduced appetite, weight loss and lethargy were the most commonly reported problems. A diagnosis was made in 184 patients, with the skin (37.90%), gastrointestinal (36.00%) and ophthalmic systems (22.00%) being most affected. The three most common diagnoses were dysecdysis (29.96%), endoparasitic burden (10.75%) and presumptive hypovitaminosis A (9.88%). Significant husbandry and signalment factors were identified in several reported disorders.

**Limitations:**

The small sample size and limited data provided in some records may have prevented the identification of potential links to specific disorders.

**Conclusion:**

This report highlights husbandry deficits that exacerbate specific disorders and emphasises the importance of owner and clinician understanding of these disorders. It serves as a foundation for future studies into how risks can be mitigated.

## INTRODUCTION

The popularity of reptiles in the UK has increased steadily.^1^ While precise statistics are difficult to ascertain, the 2023 Pet Food Manufacturers’ Association ‘Pet Population Survey’ estimated 2.4 million reptiles housed in 5% of UK households.^1^

With regards to population numbers, the bearded dragon (*Pogona vitticeps*) is closely followed by the leopard gecko (*Eublepharis macularius*). This has been attributed to their availability, inexpensive, small size, placid demeanour and range of colour variations, making them attractive options to herpetoculturists.^2^ Leopard geckos are terrestrial, crepuscular, thigmothermic, insectivorous lizards native to South and Central Asian rocky grasslands and arid plains.^2^

Despite this interest and advances in exotic medicine, research appears to be reactive to increased ownership. To date, there have been no surveys on disorders in leopard geckos in the UK. In the United States, a study was conducted on leopard geckos in 2013,^2^ but this was specifically focused on disorders of the ophthalmic system. In the years since, reptile ownership and medicine have advanced to a degree comparable to that of other non‐traditional companion animals (NTCAs), necessitating a more detailed understanding of their healthcare.

While 85% of dog and cat owners presented their animal to a veterinarian in a 12‐month period,^3^ only one‐third of reptile owners reported ever taking their animal to a veterinary clinic.^4^ Clinicians lacking confidence with reptiles have been cited as a factor influencing a culture of self‐diagnosis within herpetoculture, as well as difficulty interpreting behaviours that may indicate ill health.^4^

Therefore, this study aimed to investigate risk factors for disorders in leopard geckos in the UK to provide general practitioners with a greater understanding of disorders in leopard gecko patients and the husbandry measures their clients may implement to improve leopard gecko welfare.

## METHODS

Medical records of geckos presenting at three veterinary clinics between 1 January 2019 and 31 January 2023 were reviewed retrospectively in February 2023. The clinics were contacted for participation based on their exclusive caseload of non‐traditional companion animals, which incorporates reptiles.^5^

Two clinics used RxWorks Veterinary Software (2018), and the third used IDEXX Animana (2020). The practice management system at each practice was searched for any patients registered as ‘gecko’.

A preliminary search consisted of 248 geckos, comprising 214 leopard geckos (*E. macularius*), 28 crested geckos (*Correlophus ciliatus*), two African fat‐tailed geckos (*Hemitheconyx caudicinctus*), two gargoyle geckos (*Rhacodactylus auriculatus*), one blue‐tailed gecko (*Phelsuma cepediana*) and one peacock gecko (*Phelsuma quadriocellata*). Due to the small sample sizes and widely varying husbandry requirements, it was decided to focus the study on leopard geckos.

Inclusion criteria included leopard geckos presented with a new clinical problem in the study period, or if signs of a new problem had been observed in a veterinary surgeon consultation. Re‐visits for the same condition were recorded as one presentation. General health checks were excluded unless the clinician noted clinical problems, as this study aimed to report disorders and not the overall health status of the population.

Subsequently, the following data were recorded from each patient: species, sex, age, body condition score (BCS), presenting clinical sign(s), diagnostics performed, organ system(s) involved and final diagnosis (if stated). Individuals were categorised into five age groups: 0‒1 year, 1‒3 years, 3‒5 years, 5‒10 years and 10 years plus. Disorders were categorised into organ systems based on author interpretation of clinical notes. The final diagnoses were based on either a presumptive diagnosis written in clinical notes or confirmed based on diagnostics. As there is no validated BCS system for leopard geckos, animals were categorised as underweight, healthy or overweight based on clinical notes, which provided subjective assessments of fat coverage of the tail, and fat and muscle coverage of the limbs and spine. In some cases, a number between 1 and 5 was assigned, with 2.5‒3 being considered healthy.

Where available, data on husbandry were recorded, including heating, lighting, substrate, humidity and whether or the patient was housed in a group. Supplementary humidity was defined as enclosure misting and/or the presence of a moist substrate hide to create a localised environment of increased relative humidity. Available data on diet and supplementation were also recorded. Hard‐bodied prey was an umbrella term applied to food items that contained a mean chitin content of more than 6.00% according to a 2020 study extracting chitin from various vertebrates.^6^ The prey items recorded in this category were crickets, locusts and roaches. Prey items containing less than 6.00% chitin included mealworms, waxworms, morioworms and silkworms, and so were grouped under the term ‘worm prey’.

Data were analysed using commercially available SPSS Statistics (version 29), and a *p*‐value of less than 0.05 was considered statistically significant. Missing or unknown data were excluded from analysis. Chi‐squared tests were performed, or Fisher's exact tests if the sample size was less than 10 cases. Standard logistic regression with odds ratios and 95% confidence intervals (CIs) were calculated to evaluate potential risk factors associated with each disorder. Associations where *p*‐value less than 0.1 were entered into a multivariable univariate logistic regression model, and variables were excluded until a final minimal statistically significant model was obtained.

## RESULTS

There were 214 leopard geckos (practice one, *n* = 50; practice two, *n* = 78; practice three, *n* = 86), consisting of 114 females, 90 males and 10 of unknown sex, ranging in age from 1 month to 20.2 years (Table 1). The median age was 4 years.

**TABLE 1:** Leopard gecko disorders categorised by organ system.

	*n*	Cloaca	GI	MSK	Neuro	Ophth	Repro	Resp	Skin	Renal	Other
Total *n*		8	77	19	10	47	23	3	81	4	15
Percent of diagnoses		3.70	36.00	8.90	4.70	22.00	10.70	1.40	37.90	1.90	8.15
Age (years)											
<1	13	1	7	2	0	0	0	0	3	0	1
1 < 3	59	2	20	6	2	15	5	1	23	0	2
3 < 5	54	1	13	3	5	15	9	1	24	0	4
5 < 10	52	3	23	8	2	12	6	1	18	2	4
10 +	28	1	13	0	0	4	3	0	10	2	3
Unknown	8	0	1	0	1	1	0	0	3	0	1
Sex											
Female	114	3	37	10	7	23	**18** ^*^	**3** ^*^	44	0	3
Male	90	5	35	8	3	21	5	0	32	**4** ^*^	11
Unknown	10	0	5	1	0	3	0	0	5	0	1
BCS											
Underweight	78	1	31	8	6	15	4	1	29	3	6
Healthy	88	6	31	8	2	22	10	2	29	1	2
Overweight	25	1	9	1	0	5	6^*^	0	12	0	4
Unknown	23	0	6	2	2	5	3	0	11	0	3

*Note*: Total *n* and percent of diagnoses are greater than *n* of individual patients due to multiple organ system presentations in several patients. Statistically significant associations are denoted in bold with an asterisk (*).

Abbreviations: BCS, body condition score; GI, gastrointestinal; MSK, musculoskeletal; Neuro, neurologic; Ophth, ophthalmic; Repro, reproductive; Resp, respiratory.

Presenting signs were non‐specific in 64.02% of consultations (*n* = 137), specifically reduced/absent appetite (*n* = 80), retained shed (*n* = 47), lethargy (*n* = 34), weight loss (*n* = 34) and closed eyes (*n* = 27).

Faecal sampling was the most common diagnostic method used in 20.09% of patients (total, *n* = 43; parasitology, *n* = 38 [direct wet mounts only, *n* = 20; both wet mounts and saline/sugar flotations, *n* = 16; acid fast stain for *Cryptosporidium* spp., *n* = 2]; culture [*n* = 2, all aerobic]; and *Cryptosporidium varanii* PCR [*n* = 5, all nested]). Twenty individuals had a ‘high positive’ pinworm result recorded; however, the quantification method used was not described in many cases, preventing standardised interpretation. *Ascaris* species eggs were noted in several samples, as well as the protozoans coccidia (including *Eimeria* spp.), trichomonads and amoeba. Eleven leopard geckos were suspected to be infected with *C. varanii*, although only five were confirmed.

Diagnostic imaging was performed in 12.15% of patients (radiographs, *n* = 13; ultrasound, *n* = 13; computed tomography, *n* = 1). Blood samples were taken from 5.14% (*n* = 11). Other diagnostics included transillumination (*n* = 41), culture and sensitivity of non‐faecal material (*n* = 9), fluorescein staining of ocular lesions (*n* = 8) and fine‐needle aspirates from various masses (*n* = 4).

A final diagnosis was recorded in 85.98% of cases, with 272 diagnoses in 184 patients, many diagnosed with concurrent disorders. The most commonly affected organ systems were the skin (37.90%), gastrointestinal system (36.00%) and ophthalmic system (22.00%) (Table 1). ‘Other’ disorders accounted for 8.15% of final diagnoses (*n* = 15) and included nutritional secondary hyperparathyroidism (*n* = 8), neoplasia (*n* = 5) and septicaemia (*n* = 2).

There was a statistically significant association between sex and reproductive disorders (*p* = 0.017), with females being 3.19 times (95% CI 1.14‒8.95) more likely to present with a reproductive disorder than males. Equal numbers of pre‐ and post‐ovulatory cases were surveyed in this study (*n* = 9 each). There was also a statistically significant association between BCS and reproductive disorders (*p* = 0.026), with overweight individuals being 5.84 times (1.50‒22.81) more likely to present with a reproductive disorder than low or ideal BCS. All four individuals (100.00%) with renal disorders were male (*p* = 0.036), and all three (100.00%) with respiratory disorders were female. No other statistically significant associations were found between signalment and organ system.

The three most common diagnoses were dysecdysis (*n* = 47), endoparasitic burden (*n* = 23) and presumptive hypovitaminosis A (*n* = 19). There was a statistically significant association between age and hypovitaminosis A (*p* = 0.031), with no animals aged less than 1 year or more than 10 years diagnosed, compared to 18.64% (11/59) of individuals aged 1‒3 years, 5.55% (3/54) aged 3‒5 years, and 7.70% (4/52) aged 5‒10 years.

There was a statistically significant association between age and endoparasite presence (*p* = 0.007), with 46.15% (6/13) of individuals aged less than 1 year affected compared to 13.46% (7/52) aged 1‒3 years, 5.56% (3/54) aged 3‒5 years, 7.69% (4/52) aged 5‒10 years, and 10.70% (3/28) aged more than 10 years. No other statistically significant associations were found between signalment (including BCS) and final diagnosis.

### Husbandry factors

Heating provisions were variable, with environmental temperatures ranging from 17°C to 35°C. Heat mats were the most common heat source (*n* = 126), with other sources including lamps, ceramics and deep heat projectors. In three cases, no heating was provided. The most common primary substrate was reptile carpet (*n* = 32), followed by paper‐based substrates such as kitchen towel and newspaper (*n* = 28), sand (including ‘calcisand’) (*n* = 22) and bark/wood chips (*n* = 16). Other substrates recorded included cocoa fibre (*n* = 14), soil/compost (*n* = 14), linoleum/tiles (*n* = 5), aspen (*n* = 2) and moss (*n* = 1). Further details of husbandry and diet are shown in Table 2.

**TABLE 2:** Husbandry and diet of leopard geckos disorders categorised by affected organ system.

	*n*	Cloaca	GI	MSK	Neuro	Ophth	Repro	Resp	Skin	Renal	Other
Housed socially											
Yes	21	1	5	9^*^	1	3	2	0	10	1	1
No	169	5	65	7	5	39	17	2	63	3	10
Unknown	24	2	7	3	4	5	4	1	8	0	4
UVB provided											
Yes	77	4	37	9	3	22	7	1	39	2	5
No	90	4	24	5	5	16	13	2	23	0	6
Unknown	47	0	16	5	2	9	3	0	19	2	3
Primary substrate											
Sand (including calcisand)	22	1	9	0	2	8	1	0	14^*^	0	1
Paper towel or newspaper	28	3	12	3	0	4	4	0	8	1	2
Carpet	32	1	14	4	2	10	0	0	13	1	1
Bark or wood chip	16	1	6	2	0	3	3	0	3	0	2
Other/unknown	116	2	36	10	6	22	15	3	43	2	9
Supplemental humidity provided											
Yes	101	6	35	15	4	22	14	2	31	1	10
No	21	0	9	0	1	7	1	0	12^*^	0	1
Unknown	92	2	33	4	5	18	8	1	38	2	4
Diet											
Only worms	33	0^*^	14	1	1	12	1	1	18	0	1
Worms and hard‐bodied invertebrates	85	2	37	8	3	16	10	0	29	3	4
Only hard‐bodied invertebrates	46	5	16	3	1	9	6	1	17	0	4
Unknown	50	1	10	7	5	10	6	1	35	1	6
Supplement provided											
Yes	115	6	46	8	5	29	13	2	43	1	7
No	12	0	4	2	1	2	0	0	6	0	2
Unknown	87	2	27	9	4	16	10	1	32	3	6

*Note*: Statistically significant associations are denoted in bold with an asterisk (*).

Abbreviations: GI, gastrointestinal; MSK, musculoskeletal; Neuro, neurologic; Ophth, ophthalmic; Repro, reproductive; Resp, respiratory; UVB, ultraviolet B.

A significant association was found between leopard geckos housed with others and musculoskeletal disorders (*p* ≤ 0.001), including tissue lacerations (*n* = 12), fractures of the tail (*n* = 2), limbs (*n* = 2), jaw (*n* = 1), pelvis (*n* = 1) and spine (*n* = 1). Five fractures were subsequently attributed to nutritional secondary hyperparathyroidism. However, in the nine cases of group‐housed leopard geckos, injuries were confirmed or suspected to be due to social altercations. Those housed together (9.81%, *n* = 21) were 17.36 times (95% CI 5.50–54.74) more likely to be diagnosed with a musculoskeletal problem than those housed individually.

There was a significant association between substrate and skin disorders (*p* = 0.019), with those housed on sand (10.28%, *n* = 22) 7.58 times (95% CI 1.65–34.9) more likely to be diagnosed with a skin disorder compared to those housed on woodchip, and 4.37 times more likely (95% CI 1.32–14.45) compared to those housed on paper. There was a significant association between skin disorders and an absence of supplementary humidity (*p* = 0.021). Those not provided with supplementary humidity (9.81%, *n* = 21) were 3.01 times (95% CI 1.15–7.88) more likely to be diagnosed with a skin disorder than those provided with supplementary humidity.

There was a significant association between diet and cloacal disorders (*p* = 0.028), specifically organ prolapse through the cloaca in all eight cases. No individuals fed solely worms developing a cloacal disorder, compared to 2.35% of those fed a mix of worms and hard‐bodied food, and 10.87% of those fed only hard‐bodied food. Comparing leopard geckos where worms made up part of their diet, those fed only hard‐bodied food were 7.07 times more likely (95% CI 1.32–37.87) to be diagnosed with a cloacal disorder. No other statistically significant associations were found between husbandry or diet and organ system.

When comparing final diagnoses, a significant association was found between diet and presumptive hypovitaminosis A (*p* = 0.003). A total of 27.27% (9/33) of leopard geckos fed only worms were diagnosed with hypovitaminosis A compared to 10.59% (9/85) fed a mix of prey items and 2.17% (1/46) fed only hard‐bodied food. Those fed only worms were 18.75 times more likely (95% CI 2.26–155.35) to be diagnosed with hypovitaminosis A compared to those fed only hard‐bodied food. No other statistically significant associations were found between husbandry or diet and final diagnosis.

### Multivariable analyses

Multivariable analyses of group housing and the other variable where *p*‐value is less than 0.1 (extra humidity, *p* = 0.071) on the presence of a musculoskeletal disorder revealed that group housing remained a significant risk factor.

Multivariable analyses of sex, BCS and the other variable where *p*‐value is less than 0.1 (provision of ultraviolet B (UVB) light, *p* = 0.071) on the presence of a reproductive disorder revealed that sex and BCS remained significant risk factors.

Multivariable analyses of supplementary humidity, substrate and the other variable where *p*‐value is less than 0.1 (provision of UVB light, *p* = 0.073) on the presence of a skin disorder revealed that substrate remained a significant risk factor.

Multivariable analyses of age, diet, and the other variable where *p*‐value less than 0.1 (provision of UVB light, *p* = 0.095) on the presence of hypovitaminosis A revealed that age and diet remained significant risk factors.

## DISCUSSION

This study is the most extensive survey to date of leopard gecko disorders in the UK. The skin and gastrointestinal and ophthalmic systems were the most frequently implicated. Dysecdysis, endoparasites and hypovitaminosis A (presumptive) were the most common diagnoses.

### Dysecdysis

The integument was the body system most frequently affected, with dysecdysis being the most common disorder reported. Primary dermatitis was uncommon, and skin disorders are often secondary to underlying disease—a healthy leopard gecko will routinely ingest shed skin.^7^ Failure to do and subsequent dysecdysis has been associated with insufficient humidity and hydration, as old skin is more adhered to the fresh layer beneath.^7^ Owners often miss tourniquets of the retained shed around the digits, distal limbs and tail, causing avascular necrosis, while retained skin around the eyes leads to keratitis and conjunctivitis.^7^ In this study, the absence of supplementary humidity was associated with dysecdysis, although substrate was the most significant risk factor, with leopard geckos housed on sand most likely to exhibit dysecdysis. Previously, it has been suggested that sand assists exfoliation^8^; however, more recent literature suggests sand alone, including substitutes (such as ‘calcisand’, composed of ground limestone) irritate the skin thereby impeding ecdysis.^7^ In the wild leopard gecko terrain contains a mixture of clay, soil and small portions of sand.^2^, ^8^ As such, captive management should aim to replicate this. If this cannot be achieved then artificial and wood‐based substrates have a reduced risk of dysecdysis compared to sand alone, but wood substrates in particular have been implicated in cases of impaction.^9^

### Endoparasitic infection

Gastrointestinal disease has historically been cited as a common disorder in captive lizards,^2^, ^6^, ^7^ a finding supported by this study. Endoparasites are known to be a prominent cause of the non‐specific clinical signs such as reduced/absent appetite, weight loss and lethargy.^7^, ^9^ Such signs were commonly reported in this study, although statistical tests did not confirm a significant association with endoparasitism.

Nematodes such as ascarids and pinworms (*Oxyurid* species) are common and often non‐pathogenic in reptiles,^10^, ^11^ considered beneficial in the breakdown of cellulose and chitinous prey, aiding digestion and preventing constipation and impaction.^11^, ^12^ Clinical signs are often absent with a commensal parasite load, so clinical individuals may be subject to an advanced burden, which may even cause obstruction and cloacal prolapse.^8^, ^11^

A 2021 Brazilian study found 65.21% of asymptomatic captive leopard geckos to test positive in coproparasitologic surveys.^13^ However, the sample consisted of 23 patients housed at the same institution, which may have increased transmission risk resulting in higher rates. Among affected individuals, 93.33% exhibited *Oxyurid* eggs and 33.33% *Strongyloidea* eggs. In a survey of 331 lizards across 32 species, multiple concurrent endoparasite infections were found in the faeces of 76.10% of wild‐caught and captive‐bred specimens.^10^ While wild‐caught specimens were regarded as carrying a heavier parasite load than captive‐bred, the direct life cycle of some species promotes faster replication and reinfection in captivity. A 2013 study found that while 95.00% of 52 lizards exhibited intestinal parasites on postmortem, only 36.00% shed eggs in their faeces,^14^ emphasising that faecal results do not always correlate to parasite load. Despite this, eradicating commensals can be detrimental,^10^ so faecal egg counts are considered prudent in vivo for quantifying parasites and justifying anthelmintic treatment.^10^

This study found age to be a factor in endoparasites. Juveniles were found to harbour more endoparasites in a study^15^ on Atlas day geckos (*Quedenfeldtia trachyblepharus*). While no such association had been previously confirmed in leopard geckos, anecdotal reports suggest juveniles exhibit more noticeable parasite responses than adults due to their small size, increased stress and less developed immune status.^2^, ^10^ While statistical testing in this study did not demonstrate significance between a low BCS and endoparasites, many endoparasite‐positive individuals had a low BCS, likely a sequel of the burden rather than a risk factor, but highlights weight loss and emaciation as frequently associated signs, and may provide a basis for future studies to explore BCS as a marker for treating endoparasitism.

Studies indicate that insectivorous lizards are more susceptible to parasites than herbivorous lizards due to prey items acting as paratenic hosts to complex life cycle parasites^10^; however, diet was not found to be associated with endoparasites in this study. In agreement with previous literature, this study recommends quarantining new reptiles, practising proper hygiene, and conducting routine physical and faecal examinations.

### Hypovitaminosis A

Hypovitaminosis A can be challenging to diagnose definitively. Vitamin A is primarily stored in the liver, which can be higher risk to biopsy in small patients, making circulating blood values misrepresentative of vitamin A status.^16^ As such, diagnoses are often based on history and squamous metaplasia of the epithelium: caseous eyelid plugs, dull, dry skin and hemi‐penile plug formation in males.^16^ While the value of plasma retinol for diagnosis has been described,^17^ all 19 diagnoses in this study were made presumptively in clinical notes on the basis of such clinical signs, so overdiagnosis and misdiagnosis are possibilities.

Reptiles rely on a dietary source of vitamin A.^2^, ^8^ Feeder insects are typically deficient in vitamin A, vitamin D and calcium^17^—mealworms in particular have a very low level of vitamin A.^17^ Hence, dusting with calcium and vitamin supplements and ‘gut‐loading’ are recommended to increase nutritional value.^17^ This was performed much less often in worm‐based prey, with 35.62% of worms supplemented (*n* = 41) compared to 62.22% of hard‐bodied prey (*n* = 75). A significant number of the patients with a presumptive diagnosis of hypovitaminosis A in this study were between 1 and 3 years of age, representing the chronicity of the condition—a juvenile with suboptimal diet may take months to years to manifest clinical signs.

### Injuries

In the wild, this species rarely encounters conspecifics.^2^, ^8^ This report recorded 19 cases of musculoskeletal trauma and fractures. It was found that nearly half (47%) of fractures were due to intraspecific trauma. Therefore, it is recommended that leopard geckos be housed separately unless breeding to reduce the risk of injury.

### Reproductive disorders

Reptile reproduction is variable and complex, with females carrying most reproductive demands, intrinsically correlated to a suitable environment.^18^, ^19^ Leopard geckos are spontaneous ovulators with long active periods, typically requiring brumation to become reproductively viable.^20^ Post‐ovulatory stasis or dystocia, characterised by the failed oviposition of mature eggs, is rare in wild reptiles^20^ but common in captive lizards,^20^ which even when solitary, may produce up to eight clutches of two infertile eggs annually.^21^ Alongside vitamin A and calcium, environmental factors such as nest suitability are known to be a factor,^18^, ^19^, ^20^ but what constitutes a suitable nest for a female is speculative—wild individuals burrow in heat‐ and moisture‐retaining substrates, such as soil, leaf litter and rotting tree stumps, and even under refuse bags.^19^ Literature has linked dystocia in captive lizards to incorrect temperatures and inadequate nesting sites.^20^ Furthermore, malnourishment and the absence of UV during reproductive activity can precipitate hypocalcaemia during calcium mobilisation, affecting oviduct contraction.^22^, ^23^

Pre‐ and post‐ovulatory cases surveyed in this study exhibited broad clinical signs, including changes in appetite and behaviour, and coelomic distension. It is important to note that anorexia may be a physiological response to reproductive activity.^18^, ^23^, ^24^ A high BCS was associated with an increased risk, supporting previous claims that obesity is a factor in stasis^22^, ^23^ due to fat deposits distorting pelvic anatomy. This highlights prevention as a crucial measure through appropriate diet and the provision of suitable nest sites, such as deep substrate that promotes digging and retains heat and moisture.^23^, ^24^

### Cloacal disorders

Cloacal prolapse often with involvement of colon, oviduct or hemipenes can be secondary to excessive straining, commonly as a sequel of dystocia in females, seminal plugs in males, and trauma, infection, urolithiasis, gastrointestinal impaction and parasites in both sexes.^9^ Evidence shows worms primarily comprise collagen in contrast to the chitinous endo‐ and exoskeletons of hard‐bodied insects. A 2020 study extracted chitin from various vertebrates and found that crickets contained 5.10%‒7.30% chitin and locusts contained 14.00%‒20.50% chitin.^6^ Conversely, mealworms contained 3.00%‒4.60%, with morioworms up to 5.20%,^6^ much lower than previous study estimates.^25^ Both studies also note that chitin content varies considerably between prey species and life stage, and amino acids and cellulose also influence prey item digestibility, which would make it challenging to confirm that risk of cloacal disorder is increased by chitin alone.

In this study, none of the eight leopard geckos with a cloacal disorder was fed only worms. However, due to this small sample size, coupled with the fact that worm prey is fattening and low in calcium^7^, ^8^ and, according to our statistics, increases the risk of hypovitaminosis A, it cannot be recommended that leopard geckos be fed an exclusively worm‐based diet. Provided that husbandry parameters are optimal to ensure overall health and hydration promote normal digestion, offering a varied and supplemented diet outweighs this potential benefit of worm‐only prey.

### Ophthalmic disorders

Ophthalmic disorders were less represented in our study compared to a 2018 USA study, which found an incidence of 46.00% in 144 leopard geckos,^26^ revealing associations with older males, those without a heat source, and those lacking vitamin A supplementation. A 2020 study found that 26.92% of conjunctival swabs from 26 healthy leopard geckos returned positive results for nine bacterial species,^27^ and it should be noted that individuals without primary ophthalmic disease may exhibit ocular clinical signs secondary to another disease.

### Miscellaneous disorders

Leopard geckos show a reduced propensity for neoplasia compared to other lizards,^28^ which this study also found. Significant associations were observed between sex and renal disorders with all four being male, and respiratory disorders, with all three being female. Care should be taken not to overinterpret these small numbers, as previous research has not found a sex predilection for renal^29^ or respiratory disorders^30^ in reptiles. Further research is needed to establish true associations.

### Study limitations

There were limitations to the current study, including a small sample size, which may have been mitigated by an extended data period. However, data older than 4 years may not be most representative of current trends. While the focus of this study was practices seeing only NTCAs, more extensive studies would ideally be performed to investigate whether the disorders identified reflect those in general practice.

## CONCLUSION

This study increases our understanding of disorders in leopard geckos in the UK. The skin and gastrointestinal and ophthalmic systems were most commonly affected. The most common diagnoses were dysecdysis, endoparasites and hypovitaminosis A. Non‐specific signs, such as reduced appetite, weight loss and lethargy, were the most common signs brought to a veterinarian's attention. Numerous disorders demonstrated associations with husbandry factors, which may result in dysecdysis, suboptimal BCS and reproductive disorders. The fact that many of these conditions can be remedied, and even prevented, through species‐appropriate husbandry highlights the importance of education, resource sharing, and continued research, which would empower clinicians and their clients to promote the long‐term health and welfare of their leopard geckos.

## AUTHOR CONTRIBUTIONS

*Project concept, current literature review, data collection, introduction and discussion*: Joshua Henley. *Methodology, results and tables, and statistical analysis*: Joanna Hedley. *Provision and collation of data*: Tom Dutton. All the authors contributed to editing and review of the manuscript.

## CONFLICT OF INTEREST STATEMENT

The authors declare they have no conflicts of interest.

## FUNDING INFORMATION

The authors received no specific funding in the process or completion of this report.

## ETHICS STATEMENT

The Social Science Research Ethical Review Board at the Royal Veterinary College reviewed this study (approved: 26 January 2023, reference: SR2022‐0177).

## Data Availability

The data that support the findings of this study are available from the corresponding author upon reasonable request.
